# MEFV mutation carriage as possible predisposition factor for the development of Post Pericardiotomy Syndrome (PPS)

**DOI:** 10.1186/1546-0096-13-S1-P76

**Published:** 2015-09-28

**Authors:** ID Dechtman, I Ben-Zvi, S Yael, R Cohen, E Nachum, A Lipey, L Sternik, E Kachel, Y Kassif, A Shinfeld, D Spigelstein, J Lavee, E Raanani, A Livneh

**Affiliations:** 1 Sheba Medical Center, Internal Medicine F, Ramat-Gan, Israel; 2 Sheba Medical Center, Heller Institute of Medical Research, Ramat Gan, Israel; 3 Sheba Medical Center, Cardiac Surgery, Ramat-gan, Israel

## Background

PPS is a syndrome, which manifests with pleuropericardial inflammation and occurs in about 15-20% of patients undergoing surgery, involving the pleura, pericard or both. The pathogenesis of the syndrome is not yet fully understood. Carriage of MEFV mutations may explain the occurrence of this syndrome, which largely overlaps with FMF, in only part of the operated population.

## Goal

To determine whether MEFV mutation carriage may precipitate PPS or affect its phenotype.

## Methods

86 patients who underwent cardiac surgery were studied, 45 of whom developed PPS (study group) and 41 have not (control group). Demographic data (gender, age, region of residence, ethnic origin) and type of surgery were collected. The severity of PPS was evaluated, based on a predefined scale. Genetic analysis determining carriage of one of the three most common MEFV gene mutations (M694V, V726A, E148Q) was performed.

## Results

The rate of women was higher in the PPS group (p=0.001). No significant differences were found between the 2 groups with regards to the rate of mutation carriage. Subgroup analysis for age, ethnic origin and gender also failed to yield significant results. The severity of the PPS in carriers was lower compared to non carriers.

## Conclusions

Carriage of MEFV mutations does not predispose for the development of PPS. However carriage of MEFV mutations does affect PPS phenotype (P<0.05).

